# Comparative Veterinary Psychopharmacology in Dogs and Cats: Neurotransmitter Targets, Translational Boundaries, and Species-Specific Pharmacokinetics

**DOI:** 10.3390/ani16203228

**Published:** 2026-10-14

**Authors:** Iljin Kim, Jang-Hyuk Yun

**Affiliations:** 1 Department of Biochemistry, College of Medicine, The Catholic University of Korea, Seoul 06591, Republic of Korea; iljin.kim@catholic.ac.kr; 2 Department of Medical Sciences, Graduate School, The Catholic University of Korea, Seoul 06591, Republic of Korea; 3 College of Veterinary Medicine and Institute of Veterinary Science, Kangwon National University, Chuncheon 24341, Gangwon, Republic of Korea

**Keywords:** dogs, cats, veterinary behavioral medicine, psychopharmacology, neurotransmission, translational medicine, comparative medicine, pharmacokinetics

## Abstract

Behavioral disorders in companion animals are increasingly being managed with pharmacotherapy. However, prescription practices often rely on extrapolation from human psychiatry without considering species-specific phenotypes, diagnostic frameworks, or pharmacokinetic variability. A structured translational approach is required to align mechanistic plausibility with clinical evidence and interspecies constraints. This review consolidates the current information on the pharmacological modulation of neurotransmitter systems in canine and feline behavioral disorders using a comparative framework. Neurotransmitter circuits are mapped to relevant phenotypes. Controlled clinical evidence supports the use of selected serotonergic agents for specific chronic behavioral indications in dogs, whereas evidence in cats remains scarce and indication-specific. Controlled veterinary evidence is also available for selected agents used in acute or situational behavioral indications and for selegiline in canine cognitive dysfunction. Contrastingly, the broader neurotransmitter–phenotype relationships and emerging pharmacological targets discussed in this review are supported by mechanistic or translational evidence and should not be interpreted as established clinical indications. Cross-species translation is most defensible at the level of conserved receptors and transporters, whereas direct transfer of human diagnostic categories and subjective symptoms is limited in veterinary patients. Species- and drug-specific pharmacokinetic differences, along with limited feline data, necessitate careful dose initiation, titration, and monitoring to mitigate adverse effects and drug interactions. This review provides a structured framework for interpreting psychopharmacological evidence in dogs and cats by integrating pharmacological mechanisms, the design and scope of available clinical studies, translational boundaries, and species- and drug-specific pharmacokinetic considerations. Future research could benefit from standardized outcome measures, expanded feline trials, and pragmatic real-world designs.

## Simple Summary

Behavioral problems such as anxiety, fear, repetitive behaviors, aggression, and age-related cognitive decline can substantially affect the welfare of dogs and cats and their relationships with caregivers. Medications are increasingly being used to manage these problems, but treatment decisions are often made based on human psychiatry, which cannot always be directly applied to animals. This review summarizes and compares the current knowledge on medications used to treat behavioral disorders in dogs and cats, with a focus on how major brain chemical systems affect behavior, how strongly various treatments are supported by scientific evidence, and how dogs and cats may process medications differently. The available evidence is generally stronger for dogs than for cats, and important differences between species can affect drug safety and treatment responses. Human psychiatric research can provide useful guidance, but behavioral conditions in animals should be assessed based on observable behaviors rather than direct application of human psychiatric diagnoses. By consolidating current evidence, biological mechanisms, and species-specific differences, this review provides a practical framework for safer, more informed use of behavioral medications in dogs and cats and highlights areas where further research is needed.

## 1. Introduction

Behavioral disorders in companion animals are a considerable clinical and welfare concern in contemporary veterinary practice [1,2]. Increasing urbanization, changes in human–animal relationships, prolonged indoor housing, and extended lifespans have contributed to the heightened recognition of anxiety-related conditions [2,3], compulsive behaviors, situational fear responses, and selected aggression presentations [4,5], as well as age-associated cognitive dysfunction in dogs and cats [6]. Consequently, pharmacological intervention has become a routine component of multimodal behavior management [7].

Despite its expanding use, veterinary psychopharmacology remains characterized by heterogeneous prescription practices and evidence distribution across species and conditions [8]. In many cases, therapeutic decisions are influenced, explicitly or implicitly, by extrapolation from human psychiatric literature [9]. Although evolutionary conservation among mammalian neural circuits is broadly consistent, direct translation from human psychiatry to veterinary behavioral medicine is not straightforward [10,11]. Diagnostic frameworks in human medicine rely on subjective symptom reporting and standardized categorical criteria that cannot be applied directly in nonverbal species [10,12]. Consequently, a structured comparative approach is required to differentiate between mechanistic overlap and inappropriate diagnostic transfer [13].

In this review, we aimed to consolidate the current knowledge on neurotransmitter-targeted pharmacotherapy in canine and feline behavioral disorders via a structured comparative framework. Specifically, we aimed to (i) examine major neurotransmitter systems as potential pharmacological targets relevant to clinically observed behavioral phenotypes in dogs and cats, (ii) delineate the boundaries between mechanistic translation from human psychiatry and direct veterinary clinical evidence, and (iii) summarize the available drug- and species-specific pharmacokinetic data that are relevant to dosing, treatment duration, drug interactions, washout considerations, and safety monitoring.

**Figure 1:**
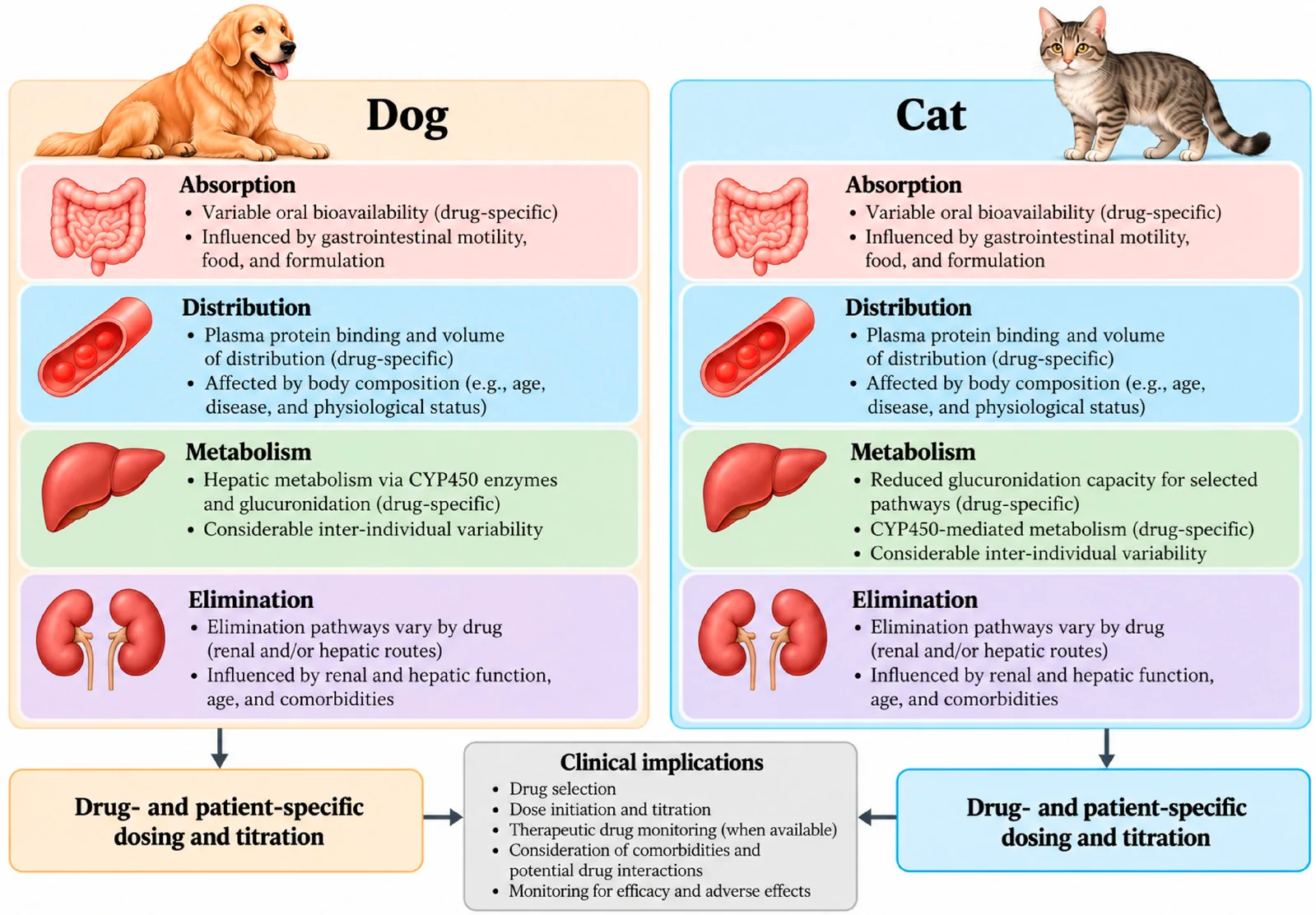
Species- and drug-specific pharmacokinetic considerations in dogs and cats. Differences in absorption, distribution, metabolism, and elimination may affect drug exposure and clinical response. These pharmacokinetic characteristics are drug- and patient-specific and should not be reduced to uniform species-wide dosing principles. Drug selection, dose initiation, titration, and monitoring should therefore be individualized based on the pharmacokinetic profile of the specific drug, patient characteristics, comorbidities, and potential adverse effects or drug interactions.

Figure 1 illustrates the key pharmacokinetic considerations in dogs and cats, including absorption, distribution, metabolism, and elimination. These processes may vary according to the drug, species, and individual patient characteristics, emphasizing the importance of drug- and patient-specific dose selection, titration, and clinical monitoring.

## 2. Methods

### 2.1. Literature Search Strategy

A structured narrative review approach was adopted to enhance transparency while maintaining the appropriate flexibility for comparative synthesis [14]. This literature search was conducted to support a structured narrative review and was not intended to be exhaustive or to meet the requirements of a systematic review. PubMed, Scopus, and Web of Science were searched for relevant literature addressing the pharmacological management and pharmacokinetics of psychotropic drugs used to treat behavioral disorders in dogs and cats. The final literature search was conducted on 2 October 2026.

The search terms were combined into four primary concept blocks: species, behavioral phenotype, pharmacological or neurotransmitter terms, and pharmacokinetic terms. Pharmacokinetic terms included “pharmacokinetics,” “bioavailability,” “absorption,” “metabolism,” “clearance,” “elimination,” “half-life,” “active metabolite,” and “drug interaction.” Terms within each concept block were combined using the Boolean operator “OR,” and relevant concept blocks were combined using “AND.” Species-related terms included “dog,” “canine,” “cat,” and “feline.” Behavioral phenotype terms included “anxiety,” “fear,” “phobia,” “separation anxiety,” “compulsive disorder,” “repetitive behavior,” “aggression,” “cognitive dysfunction,” and “noise phobia.” Pharmacological and neurotransmitter-related terms included “SSRI,” “selective serotonin reuptake inhibitor,” “tricyclic antidepressant,” “fluoxetine,” “clomipramine,” “trazodone,” “gabapentin,” “benzodiazepine,” “alpha-2 agonist,” “dexmedetomidine,” “selegiline,” “monoamine oxidase inhibitor,” “NMDA,” and “glutamate.”

Studies published between January 1990 and the final search date were considered. Earlier publications were included only when they provided foundational information directly relevant to the pharmacological mechanism, safety, or clinical use of a drug discussed in this review and when the relevant information was not adequately represented in the post-1990 literature. Only English-language publications were included; non-English publications were excluded.

### 2.2. Inclusion and Exclusion Criteria

Eligible studies included randomized controlled trials (RCTs), non-randomized prospective studies, observational cohort studies, case–control studies, retrospective studies, and case series evaluating pharmacological interventions in canine or feline clinical populations. Pharmacokinetic studies evaluating bioavailability, metabolism, half-life, clearance, elimination, or active metabolites of relevant drugs in dogs or cats were also included. Meta-analyses and consensus guidelines were included when relevant. Studies restricted to laboratory rodent models without direct veterinary clinical relevance were excluded. Opinion articles lacking supporting data were excluded unless they represented formal consensus or clinical guidelines.

Records retrieved from the three databases were combined, and duplicate records were removed before screening. Titles and abstracts were screened for relevance, followed by full-text assessment of potentially eligible articles. Reference lists of relevant reviews and included studies were also screened to identify eligible publications not captured by the database searches.

Study selection and data extraction were performed by both authors. Disagreements regarding study eligibility or interpretation were resolved through discussion and consensus.

### 2.3. Study-Design-Based Evidence Classification

To facilitate comparison of the available veterinary literature, studies were categorized based on study design. Category A included randomized controlled trials and meta-analyses; Category B included prospective non-randomized studies and well-designed observational studies; Category C included retrospective studies and case series; and Category D included expert opinion or mechanistic inference without direct clinical validation. Importantly, these categories describe the design of the available evidence rather than the overall certainty or strength of a treatment effect. They do not independently account for sample size, risk of bias, precision, consistency, duration of follow-up, or independent replication. Thus, the categories were used descriptively and interpreted along with the indication, species, study characteristics, and limitations of the supporting literature.

## 3. Neurotransmitter Systems and Behavioral Phenotypes

**Figure 2:**
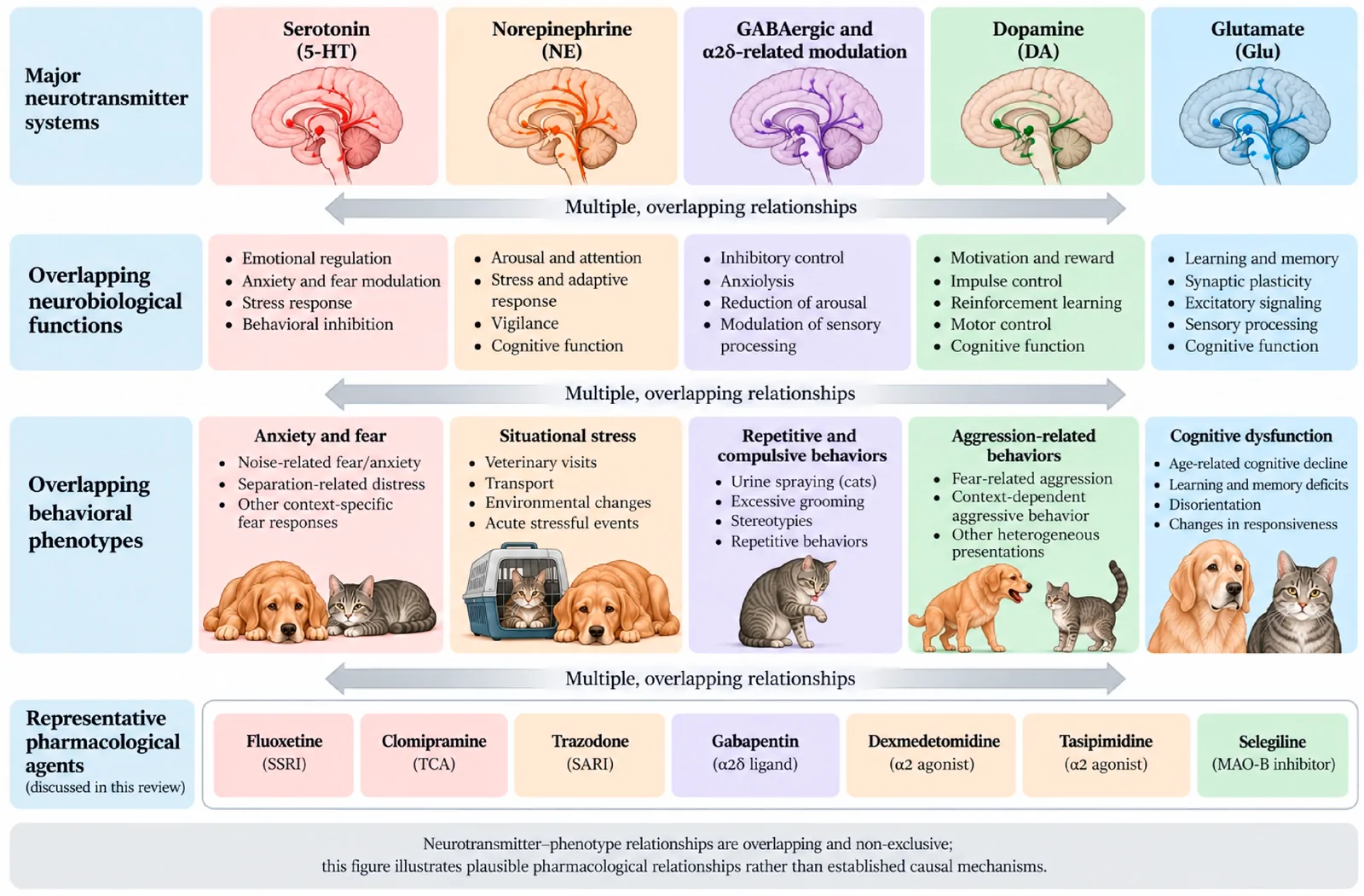
The overlapping neurotransmitter systems, neurobiological functions, behavioral phenotypes, and representative pharmacological agents discussed in this review. Major neurotransmitter systems contribute to multiple, overlapping neurobiological functions and behavioral phenotypes, and individual behavioral phenotypes may involve more than one neurotransmitter system. The pharmacological agents shown are representative drugs discussed in this review and are not intended to indicate exclusive relationships with individual neurotransmitter systems or behavioral phenotypes. The illustrated relationships represent plausible neurobiological and pharmacological associations rather than established causal mechanisms. Abbreviations: 5-HT, 5-hydroxytryptamine (serotonin); DA, dopamine; GABA, gamma-aminobutyric acid; Glu, glutamate; MAO-B, monoamine oxidase-B; NE, norepinephrine; SARI, serotonin antagonist and reuptake inhibitor; SSRI, selective serotonin reuptake inhibitor; TCA, tricyclic antidepressant.

Major neurotransmitter systems are involved in emotional processing, arousal, inhibitory control, motivation, and cognitive function and may contribute to distinct but overlapping behavioral phenotypes. Figure 2 provides an integrated overview of the principal neurobiological functions, representative pharmacological targets, and behavioral phenotypes potentially associated with these systems. Although these systems are presented individually for conceptual clarity, substantial functional overlaps exist, and many psychopharmacological agents exert therapeutic effects through multiple neurotransmitter pathways. These associations represent plausible neurobiological and pharmacological relationships and should not be interpreted as evidence that dysfunction of a single neurotransmitter system causes a specific behavioral disorder. This framework provides a basis for interpreting the following sections on individual neurotransmitter systems while distinguishing plausible pharmacological targets from demonstrated causal mechanisms. A comparative mapping of the major neurotransmitter systems across human psychiatry and canine and feline behavioral phenotypes is summarized in Table 1.

The major neurotransmitter systems involved in veterinary behavioral medicine are summarized based on their principal neurobiological functions, representative pharmacological targets, and associated clinical behavioral phenotypes. Serotonergic pathways are involved in the modulation of anxiety-related responses, compulsive and repetitive behaviors, separation-related distress, and impulse control through interactions with limbic and cortical circuits. Noradrenergic signaling originating from the locus coeruleus mediates arousal, attention, stress responsiveness, and acute fear reactions. Gamma-aminobutyric acid (GABA)ergic neurotransmission provides inhibitory control within the central nervous system and contributes to anxiolysis, sedation, and adjunctive seizure management through GABA_A_ positive allosteric modulators. Gabapentin, an α_2_δ ligand that modulates voltage-gated calcium channels, represents a mechanistically distinct anxiolytic strategy, and is therefore considered separately from direct GABAergic modulation. Dopaminergic pathways, particularly the mesolimbic system, regulate motivation, reward processing, reinforcement, and cognitive function, whereas glutamatergic neurotransmission plays essential roles in learning, memory, fear extinction, and synaptic plasticity. Representative pharmacological agents and their primary molecular targets are shown for each neurotransmitter system; however, many psychopharmacological agents affect multiple neurotransmitter pathways and behavioral domains. The level of clinical evidence is greatest in dogs, whereas evidence in cats remains limited, highlighting the need for species-specific therapeutic interpretation and further clinical investigation.

### 3.1. Serotonergic System

The serotonergic system plays a central role in anxiety regulation, impulse control, and the modulation of repetitive behaviors [15,16,17,18]. Originating primarily from the dorsal raphe nucleus and projecting widely to the limbic and cortical regions, serotonin affects emotion regulation across mammalian species.

According to controlled clinical trials in selected populations, selective serotonin reuptake inhibitors (SSRIs) and tricyclic antidepressants (TCAs) constitute first-line pharmacotherapy for separation anxiety and compulsive disorders in canine behavioral medicine [19,20]. Trazodone is also used as an adjunctive serotonergic agent in the treatment of situational anxiety and stress-related behaviors in dogs and cats, although the evidence varies by species and indication [21,22]. Evidence in feline patients remains scarce, although controlled trials support its use for selected situational indications, such as pre-visit and transport-related anxiety. However, RCTs examining chronic behavioral disorders remain sparse, and much of the available evidence is derived from small prospective studies or case series [23]. Nevertheless, RCTs have demonstrated the efficacy of fluoxetine and clomipramine in reducing urine spraying in cats [24,25]. Serotonergic agents may require several weeks to achieve maximal clinical effect. Adaptive neuroplastic changes following initial pharmacological effects have been proposed as one explanation for this delayed therapeutic response; however, this mechanism has not been fully established across species [26]. Consequently, short-acting, adjunctive agents are often administered during the initiation phase.

Potential adverse effects include gastrointestinal disturbances, altered appetite, sedation, or paradoxical excitation, and, rarely, serotonin toxicity when combined with other serotonergic agents [23,27]. Careful washout periods are required when transitioning between monoamine oxidase inhibitors (MAOIs) and selective serotonin reuptake inhibitors [28].

In the United States (U.S.), oral clomipramine hydrochloride tablets and fluoxetine hydrochloride chewable tablets are FDA-approved for the treatment of separation anxiety in dogs, with the approved use of fluoxetine including administration in conjunction with a behavior modification plan. However, many other serotonergic agents used in veterinary behavioral medicine are prescribed off-label [20,29].

### 3.2. Noradrenergic System

The noradrenergic system, primarily originating from the locus coeruleus, plays a central role in arousal, vigilance, stress responsiveness, and modulation of acute fear reactions. Increased noradrenergic activity is strongly associated with hyperarousal in human psychiatric conditions such as panic disorder and posttraumatic stress disorder [30,31]. In companion animals, noradrenergic signaling may contribute to situational fear responses, including noise-related fear/anxiety during veterinary visits, and responses to acute environmental stressors.

Alpha-2 adrenergic agonists, particularly dexmedetomidine, which are administered via oromucosal routes, have demonstrated efficacy in reducing acute noise-related fear responses in dogs under controlled trial conditions [32]. These agents reduce presynaptic norepinephrine release and attenuate sympathetic activation, providing rapid anxiolytic effects. Although clonidine has been used off-label in selected cases, published controlled veterinary clinical trials are lacking. Noradrenergic modulation is, therefore, most appropriately conceptualized as an event-targeted or adjunctive therapy rather than as a primary long-term management solution for generalized anxiety syndromes.

Cardiovascular effects, including bradycardia and hypotension, are clinically relevant considerations, particularly in geriatric and cardiac-compromised patients [33]. Species-specific dosing adjustments and careful monitoring are required to minimize hemodynamic risk. While mechanistic translation from human hyperarousal models is plausible, direct psychiatric diagnostic transfer remains inappropriate because of differences in contextual triggers and behavioral expressions.

In the U.S., dexmedetomidine oromucosal gel (Sileo) is FDA-approved for the treatment of noise aversion in dogs [32]. In 2026, the U.S. FDA approved tasipimidine oral solution (Tessie) for the treatment of noise aversion and separation anxiety in dogs [34]. The evidence supporting these approved indications includes regulatory clinical field studies submitted for product approval. Peer-reviewed evidence is also available, including a small randomized, double-blind, placebo-controlled crossover pilot study evaluating acute anxiety associated with owner departure; however, the extent of the peer-reviewed clinical literature remains limited.

### 3.3. GABAergic Agents and α_2_δ Calcium-Channel Ligands

GABA functions as the principal inhibitory neurotransmitter in the mammalian central nervous system and modulates acute anxiety responses by enhancing inhibitory tone within limbic circuits. Benzodiazepines act as positive allosteric modulators of the GABA-A receptor, enhancing inhibitory neurotransmission and providing rapid anxiolytic and muscle-relaxant effects [35]. Benzodiazepines have shown greater clinical efficacy than placebos in the short-term treatment of panic disorder and acute panic-like states, supporting their relevance in acute distress or predictable stress scenarios [36,37,38].

In veterinary patients, benzodiazepines, such as alprazolam, have been used to treat short-term situational anxiety, particularly in acute noise-related fear/anxiety and other fear/stress-related contexts [39,40]. However, concerns about tolerance, dependence, and paradoxical disinhibition, especially in impulsive or aggression-prone individuals, limit their long-term application [41,42,43]. In cats, oral diazepam is associated with rare idiosyncratic acute hepatic necrosis, but other benzodiazepines do not have the same risk profile [44]. Thus, careful drug selection and monitoring are advised in feline patients.

Imepitoin is a low-affinity partial agonist at the benzodiazepine binding site of the GABA-A receptor and is a clinically relevant non-benzodiazepine GABAergic agent [45]. In the European Union, imepitoin (Pexion^®^) is authorized for the reduction of anxiety and fear associated with noise phobia in dogs. Controlled clinical evidence supports the efficacy of imepitoin in reducing noise-related fear and anxiety in dogs [46], as summarized in Table 2.

Although not a classical GABA receptor agonist, gabapentin modulates voltage-gated calcium channels and indirectly affects excitatory neurotransmission [47]. It is commonly used to reduce situational stress in dogs and cats, particularly in veterinary clinic settings. The strength of evidence varies based on the indication and species. In feline patients, randomized placebo-controlled trials support its use in the reduction of pre-visit and transport-related stress [48]. In addition, a randomized, double-blind, placebo-controlled trial in fearful shelter cats reported that daily gabapentin, administered alongside behavioral modification, reduced stress-related measures and improved behavioral rehabilitation progress [49]. In contrast, broader applications for chronic behavioral disorders in dogs and cats rely primarily on smaller prospective or retrospective studies, supporting a moderate overall level of evidence beyond situational use [50]. Pregabalin, another α_2_δ subunit ligand, has direct clinical and regulatory evidence for its situational use in cats. Pregabalin oral solution (Bonqat^®^) is authorized in the European Union and is FDA-approved in the U.S. for the alleviation of acute anxiety and fear associated with transportation and veterinary visits in cats [51]. Its efficacy is demonstrated in prospective, placebo-controlled clinical field studies [51]. These data provide direct clinical support for α_2_δ calcium-channel ligands in feline situational anxiety and fear; however, this evidence should not be extrapolated to chronic or generalized anxiety-related conditions. GABAergic agents and α_2_δ calcium-channel ligands may provide useful support for selected acute or situational behavioral indications; however, they should be integrated into broader behavioral and environmental management frameworks rather than used as sole long-term interventions.

**Table 2:** Comparative Clinical Evidence by Species, Study Design, and Indication.

Drug/Class	Primary Target	Dogs: Clinical Evidence	Cats: Clinical Evidence
Fluoxetine (SSRI)	SERT inhibition	2 RCTs: separation anxiety [29] and compulsive disorder (*n* = 63) [19] (Category A)	1 RCT: urine spraying (*n* = 17) [24] (Category A)
Clomipramine (TCA)	5-HT and NE reuptake inhibition	1 multicenter RCT: separation anxiety (*n* = 95) [20] (Category A)	1 multicenter RCT: urine spraying (*n* = 67) [25] (Category A)
Trazodone	5-HT2 antagonism and SERT inhibition	1 randomized, double-blind, placebo-controlled crossover trial: veterinary-visit stress (*n* = 20) [21] (Category A)	1 randomized, double-blind, placebo-controlled crossover trial: pre-visit/transport anxiety (*n* = 10) [22] (Category A)
Imepitoin	GABA-A receptor modulation	1 randomized, double-blind, placebo-controlled clinical trial: noise-related fear/anxiety (*n* = 238) [46] (Category A); authorized indication in the EU	No established direct clinical evidence for behavioral indications
Dexmedetomidine	α2-adrenoceptor agonism	1 randomized, double-blind, placebo-controlled clinical trial: noise-related fear/anxiety (*n* = 182) [32] (Category A)	No comparable controlled clinical trial identified for behavioral indications; evidence remains limited/indirect (Category C–D)
Tasipimidine	α2-adrenoceptor agonism	2 pivotal randomized, double-blind, vehicle-controlled regulatory field studies: noise aversion (*n* = 160) and separation anxiety (*n* = 224) [34], plus 1 published randomized, double-blind, placebo-controlled crossover pilot study of owner-departure anxiety (Category A)	No established clinical evidence
Gabapentin	α2δ subunit of voltage-gated calcium channels	1 double-blind, placebo-controlled clinical trial: veterinary-visit stress (Category A); 1 retrospective clinical study: broader behavioral disorders (Category C)	1 randomized, blinded, placebo-controlled crossover trial: pre-visit/transport stress (*n* = 20) [48] (Category A)
Pregabalin	α_2_δ subunit of voltage-gated calcium channels	No established direct clinical evidence for behavioral indications	Placebo-controlled clinical field evidence: acute anxiety and fear associated with transportation and veterinary visits (*n* = 209) [51]; FDA-approved and authorized in the EU for this indication (Category A)
Selegiline	MAO-B inhibition	1 large prospective open-label clinical study: canine cognitive dysfunction (*n* = 641) [52], with additional experimental/behavioral studies (Category B)	No established direct clinical evidence for behavioral indications (Category D)
Benzodiazepines (e.g., alprazolam, diazepam)	GABA-A receptor positive allosteric modulation	Category B–C (situational fear/anxiety; evidence varies by agent and indication)	Category C–D; oral diazepam carries a risk of idiosyncratic acute hepatic necrosis

Category A = randomized controlled trials or meta-analyses; Category B = prospective non-randomized studies or well-designed observational studies; Category C = retrospective studies or case series; and Category D = expert opinion or mechanistic inference without direct clinical validation. Categories describe study design rather than the overall certainty or strength of a treatment effect and are indication-specific. Study numbers refer to the clinical studies directly summarized for the indicated use in this review and should not be interpreted as an exhaustive count of all publications involving each drug. For tasipimidine, regulatory field studies supporting FDA approval are distinguished from peer-reviewed clinical evidence. For studies of acute or situational stress, behavioral improvement should not be interpreted as synonymous with anxiolysis, because sedation or motor effects may reduce observable behavioral expression. Where relevant, interpretation therefore considers blinding, sedation or alertness assessments, physiological measures, and concurrent behavioral or environmental interventions. Abbreviations: 5-HT, 5-hydroxytryptamine (serotonin); MAO-B, monoamine oxidase-B; NE, norepinephrine; SERT, serotonin transporter; SSRI, selective serotonin reuptake inhibitor; TCA, tricyclic antidepressant; FDA, U.S. Food and Drug Administration; RCT, randomized controlled trial. Oral diazepam in cats has been associated with rare but potentially fatal idiosyncratic acute hepatic necrosis.

### 3.4. Dopaminergic System

Dopaminergic pathways, particularly mesolimbic circuits projecting from the ventral tegmental area to the nucleus accumbens and prefrontal cortex, affect motivation, reward processing, and repetitive behaviors. In human psychiatry, dopaminergic pathways are associated with impulse-control disorders and certain repetitive behavioral phenotypes. In veterinary medicine, dopaminergic modulation has mostly been studied in the context of canine cognitive dysfunction syndrome (CCD).

In the U.S., selegiline hydrochloride oral tablets (Anipryl^®^) are FDA-approved for the control of clinical signs associated with canine cognitive dysfunction syndrome (CDS). Clinical improvement has been reported in affected dogs, although the available evidence should be interpreted in light of the limitations of the underlying studies [52]. Improvements in cognitive and behavioral parameters have been reported; however, limitations in the study designs and variability in the reported outcomes suggest only moderate overall strength of evidence [53]. Dopaminergic contributions to compulsive behaviors and select impulsive aggression phenotypes remain mechanistically plausible but are frequently intertwined with serotonergic pathways. Consequently, attributing compulsive behaviors solely to dopaminergic imbalances oversimplifies the complexity of the relevant circuit interactions. A circuit-level model incorporating serotonergic–dopaminergic interplay could better reflect current neurobiological understanding [54].

### 3.5. Glutamatergic Pathways and Emerging Targets

Glutamatergic neurotransmission plays a critical role in synaptic plasticity, learning, and the extinction of conditioned fear responses. In human psychiatry, the modulation of N-methyl-D-aspartate (NMDA) receptors and related glutamatergic pathways has been explored in anxiety disorders and trauma-related conditions [55]. In veterinary behavioral medicine, direct clinical trials targeting glutamatergic systems are rare.

Nevertheless, mechanistic insights from translational neuroscience suggest that glutamatergic modulation enhances the efficacy of exposure-based behavioral interventions by facilitating fear extinction processes [56]. Currently, glutamatergic strategies, including NMDA receptor modulation, mGluR modulation, and riluzole, should be considered preclinical or exploratory concepts in veterinary behavioral medicine rather than established clinical treatment options for dogs and cats. Their inclusion here is intended to highlight potential directions for translational research and should not be interpreted as supporting their routine clinical use. The comparative clinical evidence for the principal pharmacological agents discussed above is summarized by the species and study-design category in Table 2.

### 3.6. Endocannabinoid System and Cannabidiol

The endocannabinoid system is increasingly being investigated as a potential modulator of stress- and anxiety-related behaviors in veterinary medicine. Cannabidiol (CBD) has complex pharmacological actions that extend beyond classical cannabinoid receptors and may affect serotonergic signaling, endocannabinoid tone, and stress-related neurobiological pathways. In dogs, controlled studies have examined CBD in models of noise-induced fear, separation-related stress, and car travel, although findings remain inconsistent [57,58,59]. A placebo-controlled study using a fireworks model did not demonstrate a clear anxiolytic effect of CBD [57], whereas a subsequent blinded study reported attenuation of selected behavioral and physiological stress measures following a single 4 mg/kg dose during separation and car travel [58]. More recent crossover data also suggest that effects may vary based on the dose, formulation, and concurrent ingredients [60]. Therefore, CBD should currently be regarded as an emerging rather than established pharmacological option for the treatment of canine behavioral disorders. Evidence regarding optimal dosing, long-term behavioral efficacy, and clinical effectiveness across behavioral phenotypes remains limited. A recent randomized, single-blind clinical trial involving 100 cats with owner-directed aggression reported reductions in aggression scores following cannabidiol (CBD) treatment, with no statistically significant differences among the active treatment groups, including fluoxetine [61]. However, these preliminary findings do not establish therapeutic equivalence or support the routine clinical use of CBD for feline behavioral disorders.

## 4. Translational Boundaries in Comparative Psychopharmacology

The central objective of comparative veterinary psychopharmacology is to delineate the boundary between mechanistic translatability and diagnostic overextension. Accordingly, translational interpretation should distinguish conservation of molecular targets and broad neural functions from experimental-model evidence, direct clinical evidence in dogs and cats, similarity of observable behavioral phenotypes, and diagnostic equivalence. While mammalian neurotransmitter systems exhibit substantial evolutionary conservation, the interpretive frameworks used to define psychiatric disorders differ fundamentally between humans and non-verbal species. This distinction has theoretical and practical implications in rational pharmacological decision-making. The principal translational boundaries between human psychiatry and veterinary behavioral medicine are summarized in Table 3.

At the mechanistic level, cross-species homology supports translational inferences. Major neurotransmitter systems are evolutionarily conserved across mammalian central nervous systems and have broadly comparable receptor families and circuit architecture [62,63,64]. These conserved elements justify hypothesis-driven therapeutic extrapolation, particularly when targeting receptor-level pharmacodynamics or neurotransmitter reuptake mechanisms. Such biological continuity forms the basis of translational psychopharmacology.

However, diagnostic equivalence does not follow from mechanistic similarity. Human psychiatric diagnoses rely heavily on subjective internal experiences, which are typically assessed using structured clinical interviews and self-report measures [65]. In veterinary medicine, behavioral syndromes are defined based on observable behavior patterns and caregiver-reported changes rather than subjective internal experiences [66]. Observable behaviors may reflect multiple underlying states, including fear, frustration, pain, cognitive decline, environmental conflicts, or learned responses. The absence of introspective reporting introduces inherent diagnostic ambiguity and limits direct categorical mapping to Diagnostic and Statistical Manual of Mental Disorders (DSM)-based constructs [67].

This distinction is particularly important because many human psychiatric categories incorporate subjective phenomena, such as intrusive thoughts, delusions, persistent worry, or alterations in thought content, which cannot be directly assessed in non-verbal veterinary patients. In dogs and cats, clinical assessment must instead rely primarily on observable phenotypes, including motor patterns, posture, vocalization, elimination behavior, avoidance, and autonomic arousal, together with contextual and caregiver-reported information. Consequently, human diagnostic labels such as post-traumatic stress disorder or generalized anxiety disorder should not be assumed to represent diagnostically equivalent conditions in companion animals. Phenotype-based descriptions of observable behavior and context provide a more defensible basis for clinical assessment and pharmacological decision-making.

Accordingly, strict transplantation of human psychiatric labels into veterinary behavioral medicine risks conceptual oversimplification. While the “anxiety spectrum” model may translate conceptually, discrete DSM diagnostic categories cannot be implemented equivalently in dogs and cats [10]. For example, a diagnosis of separation anxiety in a dog does not imply phenomenological equivalence with human separation anxiety disorder; rather, it reflects a pattern of context-specific distress behaviors observed in the caregiver’s absence. This distinction is critical when interpreting treatment response expectations and communicating prognoses to caregivers.

A circuit-level framework offers a more defensible translational model than categorical diagnostic alignment [68]. Behavioral phenotypes in veterinary patients may be interpreted in relation to multiple putative neurobiological contributors and plausible pharmacological targets, including serotonergic, noradrenergic, dopaminergic, and inhibitory neurotransmitter systems. These associations should not be interpreted as evidence that dysfunction of a single neurotransmitter system constitutes the underlying cause of a given behavioral phenotype. This circuit-informed approach reduces the risk of overgeneralization and aligns therapeutic selection with mechanistic plausibility rather than diagnostic analogy.

Furthermore, translational boundaries are not static. As objective behavioral phenotyping tools, wearable sensory devices, and standardized scoring systems evolve, veterinary behavioral diagnostics may become more granular and biologically anchored [69,70]. However, until such tools are validated across species and contexts, clinical translation must remain cautious and explicitly acknowledge areas in which mechanistic conservation does not imply diagnostic equivalence.

In summary, the most defensible translational strategy prioritizes conserved receptor and circuit mechanisms, applies phenotype-oriented interpretation, and explicitly avoids the direct replication of human psychiatric taxonomies. This calibrated framework enhances scientific rigor, while supporting rational, species-aware therapeutic adaptations.

## 5. Species-Specific Pharmacokinetics and Clinical Implications

Pharmacokinetic variability is an important translational consideration in veterinary psychopharmacology. Even when receptor-level pharmacodynamics are conserved, pharmacokinetic differences may affect drug exposure, dosing intervals, accumulation, washout periods, and the risk of adverse effects or drug interactions. Importantly, these differences are drug- and pathway-specific and should not be generalized across all psychotropic agents. Available drug-specific pharmacokinetic characteristics and their clinical implications in dogs and cats are summarized in Table 4. A feline pharmacokinetic study of compounded oral trazodone, administered alone or with gabapentin, further illustrates the importance of formulation- and combination-specific evidence when interpreting sedative effects and potential drug interactions [71].

Dogs and cats may differ in hepatic biotransformation pathways, including glucuronidation and cytochrome P450-mediated metabolism; however, the clinical relevance of these differences is drug- and pathway-specific [90,91,92]. Therefore, reduced glucuronidation capacity in cats should not be interpreted as implying uniformly slower clearance or a universal requirement for lower dosing of all psychotropic drugs. Where feline-specific pharmacokinetic data are inadequate, dosing and monitoring decisions should be based on the pharmacokinetic characteristics and therapeutic margin of the individual drug rather than on a generalized species-wide rule.

Inter-individual variability further complicates prescription. Even within species, absorption kinetics, protein binding, and metabolic rates may vary substantially. Age-related physiological changes, including reduced hepatic perfusion, reduced renal clearance, altered body composition, and polypharmacy exposure, are especially relevant in geriatric patients, who constitute a significant proportion of behavioral medicine cases [93,94,95]. However, much of the evidence regarding age-related pharmacokinetic changes is derived from human medicine, and direct pharmacokinetic data in geriatric dogs and cats remain scant; therefore, these considerations should be regarded as indirect rather than established species-specific evidence. Cognitive dysfunction syndrome, chronic anxiety, and compulsive disorders frequently occur in aging animals, highlighting the importance of pharmacokinetic vigilance.

Washout planning is an important consideration when transitioning between psychotropic drugs, particularly when monoamine oxidase inhibitors or serotonergic agents are involved [28]. Contraindicated combinations should be distinguished from drug combinations that require caution or enhanced monitoring. Appropriate washout periods are drug-specific and depend on factors including the drugs involved, active metabolites, pharmacodynamic effects, and elimination characteristics; therefore, a single washout interval should not be applied to all drug transitions. For example, when transitioning from fluoxetine to the MAOI selegiline, an extended washout period is required because of the prolonged elimination of fluoxetine and its active metabolite, norfluoxetine. At least 5 weeks should elapse between the discontinuation of fluoxetine and initiation of selegiline [96].

Polypharmacy amplifies pharmacokinetic complexity. Patients with behavioral disorders often receive concurrent medications for dermatological, endocrine, neurological, or orthopedic conditions. Cytochrome-mediated interactions, protein-binding displacement, and additive sedative effects should be carefully considered [97]. Recent canine studies have directly evaluated the concurrent administration of tasipimidine with clomipramine and fluoxetine, providing drug-combination-specific evidence that is more directly relevant to veterinary practice than predictions based solely on pharmacological mechanisms [72,88]. In feline patients, for whom interaction data are scarce, the minimization of unnecessary pharmacological layering is particularly important.

The regulatory status also intersects with pharmacokinetic interpretation. Approved indications typically reflect species-specific dose validation and safety evaluations, whereas off-label prescriptions often rely on extrapolated data and clinical experience. Off-label use is common in veterinary behavioral medicine given the limited availability of species-specific approvals. Therefore, transparent risk–benefit assessments, careful documentation, and ongoing monitoring are essential components of responsible prescription.

Ultimately, pharmacokinetic awareness transforms psychopharmacological prescription from a receptor-target exercise into a species-calibrated clinical process. Rational decision-making requires the integration of mechanistic understanding with metabolic variability, comorbidity context, age-related physiology, and practical monitoring constraints. Dose initiation, titration, and monitoring should be individualized based on the pharmacokinetic profile, therapeutic margin, indication, and available species-specific evidence for each drug.

## 6. Phenotype-Oriented Clinical Integration

A phenotype-oriented clinical decision framework integrating differential diagnoses, the intended role and duration of pharmacotherapy, species-specific evidence, adverse effects, monitoring requirements, and concurrent behavioral and environmental management is presented in Table 5.

Clinical phenotypes in this review are defined by observable behavioral patterns, their context, persistence, and functional impact rather than by direct equivalence to human psychiatric diagnoses. Separation-related anxiety/distress refers to signs of distress occurring in association with owner absence or separation, which may include vocalization, pacing, destructive behavior, inappropriate elimination, or inability to settle. Noise-related fear/anxiety is used in this review as the general descriptive term for fear- or anxiety-related behavioral responses elicited by specific acoustic stimuli, such as thunderstorms or fireworks. Terms such as “noise phobia” and “noise aversion” are retained only when they reflect the terminology used in the original study or an approved regulatory indication and should not be interpreted as interchangeable diagnostic categories. Situational fear or stress refers to context-dependent responses associated with identifiable events, including transportation or veterinary visits. Compulsive or repetitive behaviors refer to repetitive, relatively invariant behaviors that are excessive or interfere with normal function after relevant medical and environmental causes have been considered. More generalized anxiety-related presentations are used descriptively for persistent or recurrent anxiety across multiple contexts rather than as direct equivalents of human generalized anxiety disorder. In all cases, medical conditions, pain, environmental factors, and other behavioral diagnoses should be considered as potential differential or contributing factors.

### 6.1. Distinguishing Anxiolysis from Sedation and Reduced Behavioral Expression

In studies of situational stress, reduced behavioral expression should not necessarily be interpreted as equivalent to anxiolysis. Sedation, motor impairment, or reduced arousal may reduce movement, vocalization, resistance, or other observable behaviors without demonstrating a corresponding reduction in the animal’s fear- or anxiety-related state. This distinction is particularly relevant to alpha-2 adrenergic agonists, benzodiazepines, trazodone, and gabapentin. Accordingly, behavioral outcomes should be interpreted together with assessments of alertness or sedation, observer blinding, physiological measures when available, and the specific outcome measures used in each study. For example, the randomized trial of oromucosal dexmedetomidine for the treatment of noise-associated fear in dogs incorporated a functional alertness assessment and excluded observations associated with clinically relevant sedation from selected efficacy analyses. In the canine trazodone veterinary-visit trial, behavioral assessments were complemented by physiological measures, although not all physiological outcomes differed between trazodone and the placebo. In the feline gabapentin pre-visit study, reduced owner-assessed stress and improved veterinary compliance were reported, but sedation was also a common treatment effect. Thus, easier handling or reduced behavioral activity should not, in isolation, be interpreted as evidence of anxiolysis. Where pharmacotherapy was evaluated together with a structured behavior-modification or environmental-management program, the observed treatment outcome should be interpreted as evidence for the combined intervention rather than as the isolated effect of the drug.

### 6.2. Anxiety Spectrum Disorders

Chronic anxiety-related conditions, including separation anxiety and other anxiety-related phenotypes, may involve serotonergic pathways that represent plausible pharmacological targets. First-line pharmacotherapy for chronic separation anxiety typically involves SSRIs or tricyclic antidepressants [20,29]. Adjunctive agents may be used during treatment initiation or anticipated stress exposure. Behavioral modification remains an essential co-intervention for achieving durable improvement.

In cats, the evidence should be interpreted more narrowly. Controlled studies of fluoxetine and clomipramine have primarily evaluated urine spraying, whereas controlled studies of trazodone and gabapentin have focused mainly on acute transport- or veterinary-visit-related stress. Improvement in these specific outcomes should not be interpreted as establishing efficacy in the treatment of generalized chronic anxiety or compulsive disorders in cats.

### 6.3. Compulsive Disorders

Compulsive behaviors such as tail chasing, flank sucking, and excessive grooming are generally addressed using serotonergic agents, with controlled clinical evidence supporting selected compulsive disorders in dogs, and evidence in cats remaining limited [19,98]. Environmental modifications and behavioral redirection strategies are critical adjuncts.

### 6.4. Aggression

Aggression is best conceptualized as a behavioral manifestation rather than a single diagnostic entity. Clinically relevant presentations may include fear-related aggression, pain-related aggression, resource-related aggression, frustration-related aggression, and aggression associated with neurological or cognitive disease. These presentations may differ in their triggers, context, persistence, and associated motivational factors, and relevant medical and behavioral differential diagnoses should therefore be evaluated before pharmacological intervention. In particular, pain, neurological disease, cognitive dysfunction, and other medical conditions may contribute to or alter aggressive behavior. Medical assessment should extend beyond localized pain and musculoskeletal disease to consider systemic inflammatory, infectious, neurological, and endocrine conditions that may contribute to altered arousal, irritability, or behavioral reactivity [99,100]. Such conditions should be evaluated as potential contributors or confounders before attributing aggression solely to a primary behavioral disorder. Aggressive behavior should also be characterized according to its social target and situational context, including owner-directed, stranger-directed, conspecific-directed, and handling-related aggression [101,102,103]. These phenotypic distinctions may inform behavioral assessment and individualized management; however, evidence supporting specific pharmacological regimens for each aggression subtype remains limited.

Pharmacotherapy should not be considered a treatment for “aggression” as a unitary condition. Rather, medication may be used to address contributing processes such as fear or anxiety, hyperarousal, pain, impaired impulse control, or cognitive dysfunction when clinically appropriate. Therefore, treatment should be individualized based on behavioral and medical assessment and integrated with environmental management, behavior modification, and appropriate safety measures. Evidence supporting pharmacological intervention for aggression-related presentations remains limited and heterogeneous.

### 6.5. Cognitive Dysfunction Syndrome

Cognitive dysfunction in aging dogs shows a moderate response to dopaminergic modulation by selegiline [52]. The adjunctive management of anxiety, sleep disturbance, and environmental enrichment further supports cognitive stabilization.

## 7. Discussion

This review proposes a structured comparative framework for veterinary psychopharmacology that separates mechanistic translatability from diagnostic over-translation and incorporates species-specific pharmacokinetic constraints into clinical decision-making. Across neurotransmitter systems, mechanistic conservation provides a rational basis for therapeutic selection; however, the usefulness of this translation depends on the strength of evidence, phenotype validity, and safety implications of interspecies variability. Several crosscutting themes that are particularly relevant to contemporary veterinary behavioral practice and the design of future research have emerged from this comparative review.

A major limitation of the current evidence base is its marked imbalance between canine and feline studies. Controlled clinical evidence is available for selected canine indications, whereas feline evidence is more limited and indication-specific, with controlled trials concentrated in a smaller number of clinical scenarios. Consequently, extrapolation across species should remain provisional and should not be treated as equivalent evidence of efficacy or safety.

The second critical theme is the reality of polypharmacy in behavioral medicine. Long-term serotonergic agents typically require several weeks to achieve maximal clinical effect [104]. Consequently, adjunctive agents may be introduced during acute behavioral crises that require immediate risk mitigation. Situational anxiolytics, sedatives, or medications targeting comorbidities are thus often incorporated into multimodal strategies.

Although combination therapy may be rational in selected cases, it increases the complexity of causal attribution when adverse effects occur and amplifies the risk of drug–drug interactions, particularly among serotonergic agents and sedative combinations [105,106]. Polypharmacy is further complicated by concurrent medical conditions and geriatric physiology [107].

These realities underscore the clinical value of an explicit framework that links phenotypes to potentially relevant neurobiological circuits, clarifies the intended role of each agent (foundational versus adjunctive), and emphasizes washout planning and monitoring strategies. Without such a structure, prescription may drift toward incremental additions rather than a coherent regimen design, increasing the likelihood of avoidable adverse outcomes.

Aggression highlights the need for phenotype-specific clinical assessment and standardized outcome measures. Distinguishing aggression by social target, situational context, and contributing medical conditions may improve the interpretation of treatment outcomes. However, clinical evidence supporting pharmacotherapy for specific aggression subtypes remains limited, with available studies often involving small samples and selected aggression phenotypes [108,109].

This review also highlights the risk of translational misinterpretation when human psychiatric frameworks are applied to veterinary patients without explicit boundary setting. Mechanistic similarities at the receptor and transporter level can foster confidence in cross-species applicability; however, diagnostic categories and subjective symptom constructs do not translate directly [110,111]. In veterinary behavioral medicine, clinical syndromes are inferred through observable behavior patterns and caregiver reports, which can be affected by household dynamics, caregiver expectations, and contextual variability. Therefore, an overreliance on human diagnostic labels may bias clinical reasoning and lead to mismatched therapeutic targets or unrealistic expectations regarding responses. A more robust translational stance acknowledges that transferability is strongest at the mechanistic and circuit level, while phenotype definition and outcome assessment remain species- and context-specific. This distinction is particularly relevant when considering emerging targets, such as glutamatergic modulation [112]. While mechanistic plausibility is supported by human clinical data on glutamatergic modulation in treatment-resistant depression [113], direct randomized clinical trial evidence in veterinary behavioral medicine remains scarce. Thus, therapeutic implications should be framed as hypothesis-generating and situated within future research directions rather than as established clinical recommendations.

Finally, biomarker development and precision medicine approaches represent promising avenues to reduce uncertainty in psychopharmacological decision-making but require careful calibration in veterinary settings. Objective behavioral phenotyping, including quantitative measures derived from wearable devices and activity metrics, has increasingly been used to characterize psychiatric conditions and may facilitate more objective outcome assessment in clinical research [114]. Physiological or biochemical markers, such as stress-associated endocrine measures or autonomic indices, could support treatment monitoring or stratify responders versus non-responders [115]. In parallel, pharmacogenomic or metabolizer-status approaches are conceptually attractive for addressing interindividual variability and adverse event risk, particularly in cats, where pharmacokinetic data and evidence depth are limited [90,116]. However, translation of precision approaches without robust validation should be avoided and should focus on pragmatic tools that meaningfully improve decision-making rather than adding complexity without demonstrated benefits.

Overall, the available literature supports the clinical relevance of psychopharmacology for selected canine and feline behavioral indications, but the strength and generalizability of evidence vary substantially by species, drug, and phenotype. These limitations should be considered when interpreting the comparative framework presented in this review and highlight the need for more rigorous and clinically informative veterinary trials.

## 8. Future Directions

Future research in veterinary psychopharmacology should prioritize several foundational evidence gaps before more speculative precision-medicine approaches are pursued. First, standardized and clinically meaningful phenotype definitions are needed to distinguish between heterogeneous behavioral presentations and improve comparability across studies. Second, validated and species-appropriate behavioral outcome measures should be developed, with greater incorporation of blinded assessment and objective measures where feasible. Third, additional controlled clinical trials are particularly needed in cats, for which the evidence remains limited and indication-specific.

Dopaminergic and glutamatergic pathways also warrant further investigation as potential targets for behavioral disorders that respond incompletely to established pharmacotherapies. Glutamatergic modulation may be particularly relevant as an adjunct to behavioral interventions by facilitating fear-extinction learning during exposure-based protocols [117]. Dopaminergic mechanisms may also merit further investigation in compulsive/repetitive behaviors and impulsive aggression, although direct evidence supporting dopamine-targeted treatment in companion animals remains limited. However, these applications remain exploratory in companion animals and require controlled clinical validation before therapeutic recommendations can be established.

Drug-specific pharmacokinetic studies should also be expanded in both species, including repeated-dose studies and investigations in clinically relevant populations such as geriatric animals and patients with comorbidities. Longer-term studies are needed to characterize the durability of response, adverse effects, treatment discontinuation, and partial or non-response. Pragmatic studies of real-world polypharmacy should be used to evaluate commonly used drug combinations and potential pharmacokinetic and pharmacodynamic interactions. Finally, direct comparative or head-to-head trials between commonly used treatments are needed to inform relative efficacy, tolerability, and treatment selection.

Wearable devices, biomarkers, pharmacogenomics, and precision-medicine approaches may ultimately complement these priorities, but their clinical value has not yet been established. At present, these approaches should be considered exploratory research directions requiring validation rather than tools ready for routine clinical implementation.

## 9. Conclusions

Comparative veterinary psychopharmacology could benefit from this structured framework that integrates neurotransmitter targets, translational boundaries, and species-specific pharmacokinetics. Mechanistic conservation supports rational cross-species inference; however, diagnostic non-equivalence and pharmacokinetic variability necessitate cautious, calibrated application. By aligning pharmacological selection with evidence grading, circuit-level understanding, and species-aware interpretation, clinicians could improve therapeutic precision, while minimizing risks.

## Figures and Tables

**Table 1:** Neurotransmitter Systems: Comparative Translational Framework.

Neurotransmitter	Human/ Translational Context	Canine Phenotypes	Feline Phenotypes	Basis for Translation	Limitations
Serotonin (5-HT)	Anxiety, repetitive behavior, and mood regulation	Separation anxiety, compulsive disorders	Urine spraying; selected repetitive or anxiety-related behaviors	Conserved receptors and transporters; direct veterinary clinical evidence available for selected indications	Diagnostic criteria differ across species
Norepinephrine	Arousal, vigilance, and stress responsiveness	Noise-related fear/anxiety; situational fear	Acute stress responses	Conserved adrenergic targets; direct canine evidence available for selected situational indications	Context-specific triggers vary
GABA	Inhibitory control and acute anxiety responses	Acute anxiety episodes	Situational stress	Conserved inhibitory mechanisms; veterinary evidence primarily supports selected acute or situational applications	Sedation may confound behavior assessment
Dopamine	Motivation, reward processing, and impulse control	Cognitive dysfunction, compulsive/repetitive behaviors	Poorly characterized; limited clinical evidence	Conserved dopaminergic pathways; veterinary clinical evidence concentrated in canine cognitive dysfunction	Limited imaging and neurobiological data
Glutamate	Learning, memory, and fear-extinction models	Adjunct to behavior therapy (theoretical)	Emerging evidence	Conserved role in learning and synaptic plasticity; limited direct veterinary clinical evidence	Minimal direct veterinary trials

Abbreviations: 5-HT, 5-hydroxytryptamine (serotonin); GABA, gamma-aminobutyric acid.

**Table 3:** Translational Boundaries in Comparative Psychopharmacology.

Domain	Translatable	Partially Translatable	Non-Translatable
Mechanism	Receptor targets, transporters	Dose–response relationships	Exact behavioral equivalence
Diagnosis	Anxiety spectrum concept	Compulsivity models	Diagnostic and Statistical Manual of Mental Disorders (DSM)-based categorical systems
Treatment model	Drug + behavioral therapy	Treatment duration principles	Psychotherapy modality equivalence
Adverse events	GI, sedation, serotonergic toxicity	Cognitive dulling	Subjective mood reporting
Outcome measures	Behavior frequency reduction	Caregiver-reported scales	Self-reported emotional states

Abbreviations: DSM, Diagnostic and Statistical Manual of Mental Disorders; GI, gastrointestinal.

**Table 4:** Drug-Specific Pharmacokinetic and Safety Considerations in Dogs and Cats.

Drug/Species	Metabolism & Elimination	Half-Life/Active Metabolites	Safety/Clinical & Washout Considerations
Fluoxetine—Dog	Metabolized to the active metabolite norfluoxetine [72].	Repeated dosing increases fluoxetine and norfluoxetine exposure, with substantial accumulation of norfluoxetine [72].	GI signs, appetite changes, and activation/sedation may occur. Consider parent drug and norfluoxetine when switching to interacting serotonergic drugs or MAO inhibitors.
Fluoxetine—Cat	Metabolized to active norfluoxetine; oral exposure exceeds that of the transdermal formulation evaluated in cats [73].	Single oral dose: fluoxetine t1/2 ~47 h; norfluoxetine t1/2 ~55 h [73].	Prolonged parent/metabolite exposure is relevant to interactions and washout. Oral and transdermal formulations should not be assumed PK-equivalent.
Clomipramine—Dog	Hepatic/first-pass metabolism; active desmethylclomipramine is formed [74].	Single oral dose: t1/2 ~5 h; parent drug and active metabolite accumulate during repeated dosing [74,75].	TCA-related GI, sedative, anticholinergic, and cardiovascular effects may occur. Consider parent drug and metabolite during repeated dosing and switching.
Clomipramine—Cat	Desmethylclomipramine formation demonstrated in feline PK studies [76].	IV clomipramine: mean t1/2 ~12.3 h; repeated-dose feline data are limited. [76]	Species-specific safety/interaction data are limited. Do not directly extrapolate canine PK to cats.
Trazodone—Dog	Relatively rapid elimination after oral administration [77].	Single oral dose: bioavailability 84.6 ± 13.2%; Tmax 445 ± 271 min; t1/2 166 ± 47 min (~2.8 h) [77].	Sedation and GI effects may occur. Marked Tmax variability supports individualized timing for situational use.
Trazodone—Cat	Feline oral PK characterized; concurrent gabapentin may alter trazodone exposure [71].	Single oral dose: bioavailability 54.9% (7–96%); Tmax 0.17 h (0.17–0.5 h); t1/2 5.12 ± 2.56 h [71].	Sedation is clinically relevant. Lower trazodone bioavailability with gabapentin was reported in a small study; interpret cautiously.
Gabapentin—Dog	Partly metabolized to N-methylgabapentin; urinary excretion is important [78].	10–20 mg/kg PO: Tmax ~1.3–1.5 h; terminal t1/2 ~3.3–3.4 h [79].	Sedation and ataxia may occur. Rapid elimination may require repeated dosing for sustained exposure; behavioral PK/PD targets remain poorly defined.
Gabapentin—Cat	Feline single- and repeated-dose PK data are available [80].	Oral systemic availability ~89%; terminal t1/2 ~3 h in healthy cats [81].	Sedation and ataxia are clinically relevant. Consider renal function; exposure-response relationships for behavioral effects remain incompletely established.
Pregabalin—Dog	Canine PK data are available, but PK evidence specific to behavioral indications is rare [82].	Not established for the behavioral indication discussed here.	Direct behavioral-use safety data are limited. Do not extrapolate feline PK directly to dogs.
Pregabalin—Cat	Feline-specific PK data are available for pregabalin oral solution [83].	At 5 mg/kg PO, mean t1/2 was 14.7 h; exposure after re-dosing at 24 h was comparable with single-dose exposure [83].	Sedation, ataxia, and lethargy are clinically relevant; GI effects may occur. Consider potential additive effects with other sedating drugs.
Dexmedetomidine—Dog	PK is strongly dependent on the route of administration [84].	Oral-transmucosal: bioavailability 11.2 ± 4.5%; Tmax 73 ± 33 min; terminal t1/2 152 ± 146 min; IV t1/2 36 ± 6 min [84].	Sedation, bradycardia, and hypotension are important. Do not extrapolate IV/IM PK directly to oral-transmucosal use; assess cardiovascular risk/contraindications.
Dexmedetomidine—Cat	Feline PK has been characterized after parenteral administration [85].	After IM administration, rapid absorption and t1/2 ~1 h have been reported [85].	Sedation and cardiovascular effects are clinically important. Comparable feline oral-transmucosal behavioral-use PK data are lacking.
Imepitoin—Dog	Extensively metabolized, with metabolites predominantly excreted via the fecal route [86].	Terminal plasma t1/2 was approximately 1.5–2.0 h in healthy Beagle dogs; plasma t1/2 may not directly reflect the duration of pharmacological activity [86].	Generally well tolerated; neurologic/sedative effects may occur. Consider drug-specific interactions with other centrally acting agents.
Imepitoin—Cat	Feline PK has been characterized after repeated oral administration [87].	Cmax, AUC, and Tmax have been reported, but terminal t1/2 was not established [87].	Behavioral-use safety and efficacy data remain limited; do not directly extrapolate canine behavioral-use data to cats.
Tasipimidine—Dog	Canine PK data are available from single- and repeated-dose studies [88].	30 μg/kg PO: Tmax ~1 h; t1/2 ~1.6 h after a single dose and ~1.8 h after repeated BID dosing [88].	Sedation/cardiovascular effects may occur. Repeated clomipramine coadministration increased tasipimidine exposure [88].
Tasipimidine—Cat	No established species-specific clinical PK data identified.	Not established.	Safety/PK for behavioral use not established; direct extrapolation from canine data is not justified.
Selegiline—Dog	Low oral bioavailability has been demonstrated [89].	Single oral dose: bioavailability 8.51 ± 3.31%; Tmax 25 ± 5.8 min; terminal t1/2 60.2 ± 9.6 min [89].	Important serotonergic/monoaminergic interactions must be considered. Plasma t1/2 alone should not determine washout; consider active metabolites, pharmacodynamics, and the interacting drug.
Selegiline—Cat	Species-specific clinical PK data are extremely rare.	Not established.	Feline behavioral-use safety data are scarce; canine PK should not be directly extrapolated to cats.

Cats have reduced glucuronidation capacity for selected metabolic pathways; however, the clinical relevance is drug-specific. Reduced feline glucuronidation should not be interpreted as implying uniformly slower clearance or a universal requirement for lower dosing of psychotropic drugs. Where glucuronidation is not a major disposition pathway for a specific drug, species differences should be interpreted using drug-specific pharmacokinetic data. Abbreviations: BID, twice daily; GI, gastrointestinal; IM, intramuscular; IV, intravenous; MAO, monoamine oxidase; PK, pharmacokinetic; PO, oral administration; TCA, tricyclic antidepressant; Tmax, time to maximum plasma concentration; t1/2, elimination half-life.

**Table 5:** Phenotype-Oriented Clinical Decision Framework.

Phenotype	Key Differentials	Medication Role	Use	Species-Specific Evidence	Common AEs	Monitoring/Concurrent Management
Separation-related distress (dog)	Medical causes; confinement/social factors	Adjunct to behavior modification	Long-term	Fluoxetine/clomipramine; controlled canine trials (Category A)	GI signs; appetite change; sedation/excitation	Monitor response/AEs and drug interactions; concurrent behavioral and environmental modification
Noise-related fear/anxiety (dog)	Pain; other fear triggers	Event-targeted adjunct	Situational	Dexmedetomidine/tasipimidine; controlled canine evidence (Category A)	Sedation; bradycardia; hypotension	Consider canine route-specific PK and individual cardiovascular risk; distinguish sedation from anxiolysis; environmental control
Compulsive/repetitive behavior (dog)	Dermatologic; neurologic; pain; stereotypy	Adjunct after differential assessment	Long-term	Controlled canine evidence supports selected serotonergic approaches (Category A)	GI signs; appetite change; sedation/excitation	Monitor target behavior/AEs; evidence is primarily canine and phenotype-specific; combine with behavioral modification
Urine spraying (cat)	Lower urinary tract disease; litter-box factors; social/territorial stress	Adjunct to environmental/behavioral management	Long-term/indication-specific	Fluoxetine/clomipramine; controlled feline trials specifically for urine spraying (Category A)	GI/appetite changes; sedation/excitation	Monitor spraying frequency/AEs; evidence should not be generalized to broader chronic anxiety disorders; address environmental contributors
Pre-visit/transport stress (cat)	Pain/illness; handling sensitivity; prior aversive experience	Pre-event adjunct	Situational	Trazodone/gabapentin/pregabalin; controlled feline evidence for situational use (Category A)	Sedation; ataxia; other agent-dependent effects	Assess sedation separately from fear reduction; use feline drug-specific PK and low-stress handling/carrier acclimation
Cognitive dysfunction-associated behavioral changes (dog)	Medical; neurologic; sensory; pain-related causes	Adjunct after diagnostic assessment	Long-term	Selegiline; prospective/observational canine evidence (Category B)	Agent-specific AEs/interactions	Monitor response, tolerability, and concurrent medications; canine use is better established than feline use; environmental enrichment

Study-design categories: Category A = randomized controlled trials or meta-analyses; Category B = prospective non-randomized studies or well-designed observational studies; Categories describe the study design rather than the overall certainty or strength of a treatment effect. Clinical scenarios were selected to represent commonly studied canine and feline behavioral presentations with direct clinical evidence for the pharmacological strategies discussed in this review, while providing representation of both species. Medication should be integrated with appropriate medical assessment and concurrent environmental and behavioral management. Dose initiation and titration should be individualized based on the specific drug, indication, formulation, patient characteristics, clinical response, and adverse effects. Abbreviations: AEs, adverse effects; GI, gastrointestinal.

## Data Availability

No new data were created or analyzed in this study. Data sharing is not applicable to this article.

## Abbreviations

The following abbreviations are used in this manuscript:

CCD	cognitive dysfunction syndrome
DSM	Diagnostic and Statistical Manual of Mental Disorders
EMA	European Medicines Agency
FDA	U.S. Food and Drug Administration
GABA	gamma-aminobutyric acid
GAD	generalized anxiety disorder
MAOI	monoamine oxidase inhibitor
MAO-B	monoamine oxidase-B
NE	norepinephrine
NMDA	N-methyl-D-aspartate
OCD	obsessive–compulsive disorder
PTSD	post-traumatic stress disorder
RCT	randomized controlled trial
SERT	serotonin transporter
SSRI	selective serotonin reuptake inhibitor
TCA	tricyclic antidepressant
5-HT	5-hydroxytryptamine (serotonin)
